# A remote care navigation solution associated with improved utilization and outcomes of mental healthcare: A nationwide cohort study in the USA

**DOI:** 10.1371/journal.pone.0331454

**Published:** 2025-09-18

**Authors:** Emily J. Ward, Matt Hawrilenko, Geetu Ambwani, Millard Brown, John H. Krystal, Philip R. Corlett, Adam M. Chekroud

**Affiliations:** 1 Spring Health, New York, New York, United States of America; 2 Yale University, New Haven, Connecticut, United States of America; Taipei Medical University, TAIWAN

## Abstract

**Objective:**

To examine how clinical care navigation–assistance in accessing healthcare and social services–relates to mental healthcare utilization and clinical outcomes, and whether effects are consistent for people of color.

**Methods:**

This retrospective cohort study included participants using a digital mental health benefit (Spring Health), sponsored by 2,045 US employers from 2018−2023. Participants had access to therapists and Care Navigators, clinicians who help select treatment options and schedule appointments. Primary measures were care utilization (conversion to care, multiple-session attendance) and clinical effectiveness (treatment duration, PHQ-9 depression scale, GAD-7 anxiety scale).

**Results:**

36,964 participants had at least 1 mental health assessment and complete demographic information. 13,122 participants who used care navigation were matched to 23,842 participants who did not with 1:2 propensity score matching using demographic and clinical characteristics. Care navigation was associated with increased therapy utilization (OR, 7.10; 95% CI, 3.36–15.00, P < 0.001), multiple-session attendance (OR, 1.57; 95% CI, 1.46–1.69, P < 0.001), number of treatment sessions (IRR, 1.36; 95% CI, 1.33–1.39, P < 0.001), additional clinical improvement (depression: 0.93 points, 95% CI, 0.11–1.75; anxiety: 0.87 points, 95% CI, 0.12–1.62) compared to therapy alone for participants with severe baseline symptoms. White participants and participants of color had similar outcomes.

**Conclusions:**

Participants using care navigation had improved mental healthcare utilization, retention, and reduced depression and anxiety, which was consistent for people of color. Clinical implementation of care navigation may be associated with greater engagement in care, a key requisite for improving treatment outcomes.

## Introduction

People face multiple barriers to obtaining mental healthcare [1] resulting in unmet treatment needs [2–5]. Consequently, mental health disorders are the leading cause of disability worldwide [6]. Barriers include psychological barriers (e.g., stigmatization, perceived ineffectiveness, privacy concerns) and practical barriers (e.g., inability to pay, not knowing where to go, lack of childcare or transportation) [7]. In the US, these barriers are larger for groups disproportionately affected by systemic racism, such as Black, Latinx, and Asian individuals [8,9].

Providing assistance to individuals coordinating health and social care services may improve access to and outcomes in mental health treatment. One such model is care navigation, where a care navigator (licensed clinician, trained peer, or combined team) provides information and guidance to help patients’ make appropriate choices about their health. Care navigation is associated with increased access to healthcare [10] and health insurance (gaining and retaining insurance [11,12]), especially among those that have historically experienced discrimination, and with positive care outcomes, especially in domains such as cancer care, which entails many treatment options. Because mental healthcare also involves varied treatment options and subspecialties, care navigation may be effective in addressing barriers to mental healthcare. [13]

Care navigation has shown promising results in several mental health contexts. In severe cases of mental illness, peer and community-based care navigation helps patients connect to primary care after a psychiatric crisis [14], improves healthcare appointment scheduling and attendance [15,16], promotes recovery [17], and improves quality of life and general and psychological health. [17] In the general population, care navigation provided by licensed providers coupled with screening and outreach improves clinical and workplace outcomes for depressed employees. [18] However, care navigation has only been evaluated in limited settings and the median number of participants is around 60 [19], with the largest studies consisting of approximately 1800 children [20] or approximately 600 working adults [18]. Racial and ethnic minority groups are historically less likely to seek mental healthcare and more likely to drop out of treatment early [7,21,22] potentially due to lower perceived need for treatment compared to White people and more negative beliefs about treatment [23, 24]. Yet, small peer and community-based care navigation programs have helped mitigate such disparities by improving access to primary care clinics [25] and increasing use of mental healthcare [15–17].

Our retrospective cohort study is the largest-ever evaluation of care navigation, here evaluated within an employer-sponsored mental health program [26], with over 20 times the number of participants as the next largest study and over 35,000 more participants than the previous study of the mental health program [26]. Our primary objective examined whether therapy utilization and clinical improvement in depression and anxiety was associated with care navigation use. A secondary objective determined whether these effects were consistent for people of color.

## Methods

This study followed the Strengthening the Reporting of Observational Studies in Epidemiology (STROBE) reporting guideline for observational studies. The Yale Institutional Review Board approved the study (IRB protocol ID: 2000029276) and determined it was not human participant research and did not require informed consent. Data were accessed for research purposes 10/10/2023.

### Program design

The study analyzed data from a digital mental health program (Spring Health; Spring Care Inc), sponsored by 2,045 US employers, which has demonstrated improvement in clinical and financial outcomes. [26]

Enrolled individuals accessed program components accessed through a single, secure mobile application and/or web platform, which integrated all clinical and operational services (assessment, scheduling, provider notes, and billing) to support measurement-based care. Upon enrollment, individuals voluntarily completed standardized self-report measures, including the (Patient Health Questionnaire-9 [PHQ-9]; range, 0–27) [27,28] the Generalized Anxiety Disorder-7 [29], and functional impairment (Sheehan Disability Scale [30]) to identify each individual’s specific needs. An overall risk level was assigned according to the most severe score on any instrument: PHQ-9 or GAD-7 scores >=15 signified high risk, 10–14 moderate risk, 5–9 moderate-low risk, and <5 low risk. They indicated their optimism for therapy’s effectiveness and selected regular intervals for follow-up assessments. Assessment results generated a personalized care plan that guided subsequent care recommendations.

Care recommendations ranged from using a library of digital self-help modules (e.g., mindfulness exercises, CBT skill-builders) or coaching for low to low-moderate risk concerns. For moderate to high risk concerns, participants could engage in unlimited, no-cost video appointments with care navigators (see Care Navigation) who assisted with finding appropriate care, or could browse the network of clinical care providers and self-schedule appointments directly. Regardless of risk level, all participants had access to free or low-cost access to psychotherapy (with additional sessions offered as an in-network benefit covered by their health plan) and medication management through video or in-person sessions. Providers listed their clinical specialties and modalities on their profiles, with the predominant modality being cognitive behavioral therapy. Across these different care options, the platform integrated utilization reporting and outcome tracking to support measurement-based care.

### Care navigation

All care navigators held master’s degree-level clinical licenses. As part of their degree programs, licensure requirements, and ongoing continuing-education units, care navigators completed foundational diversity and cultural-competence training designed to recruit and develop a diverse workforce, ensure counselors have a solid knowledge base of different cultures, and foster skills in addressing the needs of varied populations with sensitivity to social diversity and systemic oppression. Beyond these baseline requirements, care navigators within the program underwent specialized instruction tailored to their role. This included training in member needs assessment, risk evaluation, care coordination, and safety planning—with every module grounded in principles of culturally responsive and inclusive practice.

Within the program, care navigation was delivered by phone and frequently served as the first clinical point of contact for individuals, enabling them to help destigmatize mental health care and reduce barriers to accessing support. Care navigators leveraged clinical training to meet individuals at a critical point in their mental health journey, and guided them toward appropriate, effective care: they discussed assessment outcomes, helped select treatment options, identified appropriate therapists, and scheduled appointments.

All enrolled individuals could use care navigation, but the recommendation provided through the digital platform could differ: high-risk individuals (see Program Design) always received the recommendation, while lower risk individuals’ recommendations depended on date of enrollment and their specific mental health difficulties (see Program Design in Online Supplement). Individuals could schedule unlimited free appointments with care navigators.

### Cohort study design

We conducted a retrospective cohort study comparing two key groups: participants who used care navigation and those who did not. This was an open cohort for individuals enrolled in the program between January 1, 2018 and May 31, 2023.

### Inclusion criteria

4,645,571 people (employees and dependents) were eligible for the benefit and 440,199 enrolled (9.5% enrollment rate). From those enrolled, individuals were selected for the current study if they were over 18 years at enrollment, were in the U.S., had taken at least 1 assessment between January 1, 2018 and May 31, 2023, provided an optimism rating and complete demographic information (age, race, gender).

### Measures

#### Utilization of care.

*Conversion to care*, determined once a participant attended an appointment (psychotherapy, medication management, coaching, but not care navigation) and *multiple session attendance,* determined once a participant attended a second appointment (excluding care navigation) were outcome variables. *Duration of care* was the number of appointments (excluding care navigation) within the study period with no more than 90 days between appointments. We compared participants who interacted with a care navigator *before* their first clinical appointment to those who did not to determine the effect on conversion to care, and those who interacted with a care navigator at any point to those who did not to determine the effect on multiple session attendance and duration of care.

#### Depression and anxiety.

*Depression symptoms* were measured with PHQ-9 [27] (nine-item assessment) and *anxiety symptoms* were measured with GAD-7 [29] (seven-item assessment), with both measuring symptom frequency, scored 0–3 (*not at all* to *nearly every day*). Assessments were elective and were solicited at a frequency chosen by participants. PHQ-9 and GAD-7 scores were outcome variables for assessing clinical improvement, and covariates for predicting conversion to care, multiple session attendance, and duration of care. *PHQ-9 baseline severity* (range 0–27, mean = 11.1, SD = 6.2) and *GAD-7 baseline severity* (range 0–21, mean = 10.0, SD = 5.6) were the scores from the assessment(s) taken immediately prior to starting therapy (if participants started therapy) or at the initial assessment date (if they did not start therapy).

#### Factors associated with utilization of care and clinical improvement.

The initial assessment also measured *treatment optimism* (self-report, range 1–10 [mean = 7.0, SD = 2.1]). A *recommendation for care navigation* (yes/no; 14.3% of participants received the recommendation) was part of participants’ personalized care plan (See Program Design). *Age* at program enrollment ranged 18–77 (mean = 36.2, SD = 10.5), *person of color* was classified as two broad categories (white/person of color; percent white: 59%), except for subgroup analyses were *race* had seven categories (asian, black, latinx/hispanic, middle eastern, mixed-race, native american, white). *Gender* was classified as male vs. non-male (percent male: 26.9%). The *number of employer-sponsored sessions* ranged from 0–12 (mean = 7.0, SD = 3.1). *Therapy session number,* excluding care navigation sessions, was included when modeling clinical improvement.

### Statistical analysis

Propensity score matching was used to estimate the effect of care navigation on utilization of therapy and clinical outcomes by matching participants who used care navigation to those who did not. The propensity score was estimated using logistic regression based on baseline severity (PHQ-9 and GAD-7 scores), optimism for treatment, age, person of color, gender, number of sponsored therapy sessions, and care navigation recommendation. 1:2 (care navigation to no care navigation) nearest-neighbor matching was used, with poor matches excluded prior to analyses. Good balance was achieved between the two groups, with all standardized mean differences below 0.02 after matching, except for care navigation recommendation (std. mean difference = 0.3).

Group differences in therapy utilization and clinical outcomes were estimated using multiple regression models with a linking function to accommodate different outcomes: a logit link was used for categorical outcomes (conversion to care, multiple session attendance), a negative binomial model with a log link for count data (number of sessions), and an identity link for continuous outcomes (depression, anxiety). When repeated observations were measured for the same participants (such as for depression and anxiety scores), we used a mixed-effects model where repeated observations (level 1) were nested within participants (level 2), and random intercepts and time effects were included at level 2. To control for unmeasured employer effects and for variability in approach or skill, employer and care navigator were also included as random intercepts at level 2. For employers managed by Professional Employer Organizations (PEO; n = 1652), these companies were grouped by their PEO (corresponding to a total of 393 unique employer ids included as random effect terms in these analyses). In the negative binomial model for number of sessions, the random effects for employer and care navigator were omitted to allow the model to converge. Clinical rates of improvement were estimated using a cubic polynomial with number of treatment sessions as a level 1 covariate. Improvements rates were transformed to total improvement from baseline to treatment endpoint using the delta method. The delta method multiplies the average number of sessions by group (i.e., the average endpoints) by the rate of change given by linear, quadratic and cubic coefficients and by any relevant interaction coefficients (i.e., uses care navigation x session, etc). The delta method obtains standard errors from a function that is a combination of model parameters. Cohen *d* effect sizes for clinical improvement were calculated by dividing the overall effect size by the baseline SD with commonly used thresholds to categorize effects as small (*d* < 0.50), medium (*d* < 0.8), and large (*d* > 0.8).

To determine which factors were associated with therapy utilization, the set of covariates described above (baseline severity, optimism about treatment, care navigation recommendation, age, gender, person of color, number of sponsored sessions) were modeled as main effects, along with an interaction between care navigation use, baseline severity and person of color (care navigation use × baseline severity x person of color) to account for different effects among participants with different symptom severity and identification as a person of color. The primary outcomes of interest were the main effect of care navigation, the interaction between care navigation use and baseline severity, and the interaction between care navigation and person of color. Clinical improvement was modeled with therapy session number as a time-varying covariate along with the same covariates, except that baseline severity (PHQ-9 baseline severity when modeling depression and GAD-7 baseline severity when modeling anxiety) was now included as an outcome, corresponding to session number 0. The primary outcome of interest was to show whether total improvement during therapy was greater for those using care navigation.

Because the primary variable – use of care navigation – was included in higher-order interactions (e.g., two- and three-way interactions between care navigation use, baseline severity scores, and person of color), all variables were mean-centered prior to analyses. Thus, the main effects of care navigation can be interpreted as the effect using care navigation for those with the average baseline severity and undefined (average) identification as a person of color.

All statistical tests were 2-sided with significance set at α = 0.05. All analyses were conducted in R 4.2.1.

## Results

### Participant characteristics

74,795 participants amongst 2,045 employers across all 50 US states met inclusion criteria. 14,946 (20.0%) participants used care navigation and 59,849 (80.0%) did not. 23,842 participants without care navigation were matched to 13,122 participants using care navigation through propensity score matching (see Propensity Score Matching), for a total sample size of 36,964 (6.5% dependents). These two groups were well balanced on PHQ-9 and GAD-7 severity, optimism for treatment, age, person of color, gender, and total employer-sponsored visits (Table 1). However, significantly more participants in the care navigation group were recommended care navigation compared to the group without care navigation (22.0% vs. 9.9%, P < 0.001).

**Table 1:** Characteristics of exposure groups after propensity score matching.

Characteristic	No Care Navigation	Care Navigation	p-value^2^
	N = 23,842^1^	N = 13,122^1^	
**PHQ9 baseline severity**			0.5
Mean (SD)	11.1 (6.2)	11.1 (6.4)	
Median [Min, Max]	11.0 [0.0,27.0]	10.0 [0.0,27.0]	
**GAD7 baseline severity**			0.5
Mean (SD)	10.0 (5.6)	10.0 (5.5)	
Median [Min, Max]	9.0 [0.0,21.0]	9.0 [0.0,21.0]	
**Optimism for treatment**			0.3
Mean (SD)	7.04 (2.05)	7.06 (2.05)	
Median [Min, Max]	7.00 [1.00,10.00]	7.00 [1.00,10.00]	
**Race**			>0.9
asian	2,481 (11%)	1,387 (11%)	
black	3,932 (17%)	2,261 (17%)	
latinx/hispanic	2,723 (12%)	1,532 (12%)	
middle eastern	14 (<0.1%)	10 (<0.1%)	
mixed-race	496 (2.2%)	295 (2.2%)	
native american	21 (0.1%)	17 (0.1%)	
white	14,175 (58%)	7,620 (58%)	
**Age**			>0.9
Mean (SD)	36 (10)	36 (10)	
Median [Min, Max]	34 [18,78]	34 [18,76]	
**Gender**			>0.9
female	17,384 (72%)	9,436 (72%)	
male	6,341 (27%)	3,598 (27%)	
other	117 (0.7%)	88 (0.7%)	
**Total sponsored visits**			0.13
Mean (SD)	7.0 (3.1)	7.1 (3.1)	
Median [Min, Max]	6.0 [0.0,12.0]	6.0 [0.0,12.0]	
**Recommended Care Navigation**			<0.001
Not recommended	21,498 (90%)	10,194 (78%)	
Recommended	2,344 (9.9%)	2,928 (22%)	

^1^n (unweighted) (%).

^2^Wilcoxon rank-sum test for complex survey samples; chi-squared test with Rao & Scott’s second-order correction.

### Clinician characteristics

A total of 2,659 healthcare clinicians, including 107 Care Navigators, participated over the study duration. 90% of Care Navigators were female, and 24% of Care Navigators were White. The top licenses among Care Navigators were LCSW, LPC, and LMFT, with 88% of Care Navigators holding at least one of these licenses.

### Use of mental healthcare

**Fig 1:**
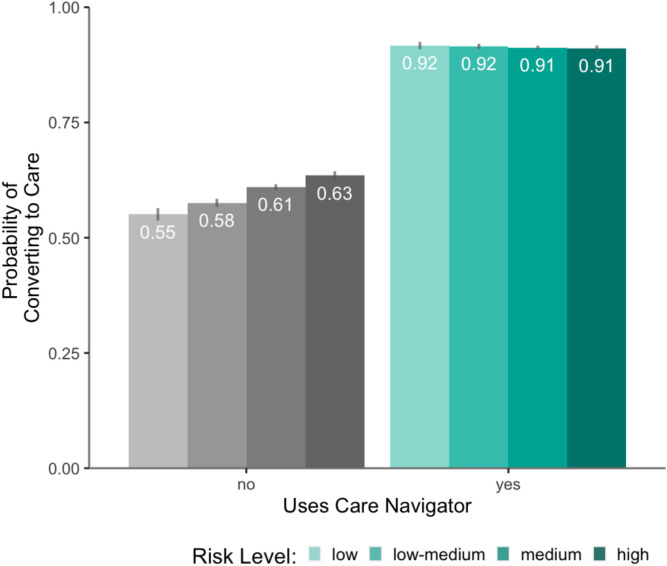
Conversion to care with care navigation. Probability of converting to care associated with using care navigation, as function of symptom severity at baseline. Error bars correspond to 95% prediction intervals.

**Fig 2:**
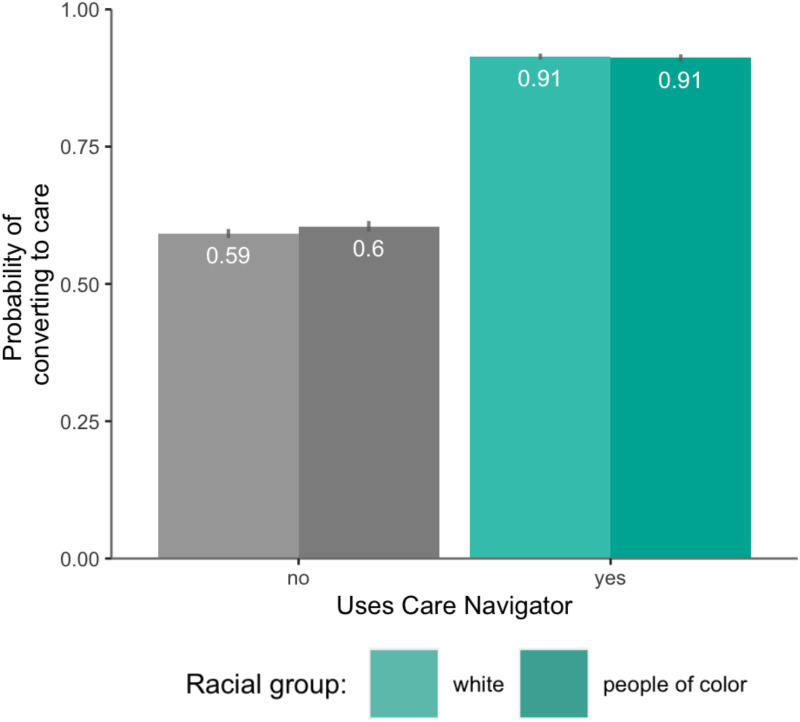
Conversion to care with care navigation for white participants and participants of color. Probability of converting to care associated with using care navigation (among participants who provided their race) for white (lighter bars) participants and participants of color (darker bars). Error bars correspond to 95% prediction intervals.

Participants who used care navigation had much higher odds of starting therapy than those who did not (OR, 7.1; 95% CI, 3.36–15.00, P < 0.001; S1 Table in S1 File), with 87.2% converting to care in the care navigation group compared to only 50.8% in the group without it. Baseline symptom severity positively predicted conversion to care (OR, 1.01; 95% CI, 1.007–1.017, P < 0.001), but less strongly for patients using care navigation (OR, 0.975; 95% CI, 0.965–0.985, P < 0.001) (Fig 1). Identifying as a person of color had no main effect (OR, 1.02; 95% CI, 0.968–1.083, P = 0.412), and care navigation did not interact with identifying as a person of color (OR, 0.92; 95% CI, 0.809–1.005, P = 0.354) (Fig 2). There was no interaction between care navigation, person of color and baseline symptom severity (OR, 0.996; 95% CI, 0.975–1.017, P = 0.688). When analyzed by specific racial groups, there was no interaction between race and care navigation on conversion to care relative to White participants (Ps > 0.072), except for Native American participants (n = 38), whose odds of converting to care with care navigation were significantly less than the odds for White participants (OR=0.21, 95% CI 0.05–0.81, p < 0.023).

Participants who used care navigation had greater odds of attending more than one therapy session (i.e., less likely to drop out after a single session) than those who did not (OR, 1.57; 95% CI, 1.463–1.688, P < 0.001; S2 Table in S1 File), with 84.4% attending multiple sessions in the care navigation group compared to 78.8% in the group without it. Baseline severity had a main effect (OR, 1.007; 95% CI, 1.000–1.013, P = 0.035) and interacted with care navigation (OR, 1.02; 95% CI, 1.005–1.029, P = 0.005): for participants not using care navigation, the odds of attending more than one session decreased with baseline severity, whereas for participants using care navigation, the odds of attending more than one session increased with baseline severity. There was no main effect of identifying as a person of color (OR, 0.938; 95% CI, 0.874–1.008, P = 0.081). Care navigation was equally associated with increasing multi-session attendance for White participants and participants of color (OR, 1.016; 95% CI, 0.884–1.167, P = 0.828), and did not interact with identifying as a person of color and baseline symptom severity (OR, 1.015; 95% CI, 0.991–1.039, P = 0.225). When analyzed by specific racial groups, there was no interaction between race and care navigation on attending multiple sessions relative to White participants (Ps > 0.423), except for mixed race participants (n = 515), whose odds of attending multiple sessions with care navigation were significantly greater than the odds for White participants (OR=1.93, 95% CI 1.15–3.23, P = 0.012).

### Overall clinical outcomes

**Fig 3:**
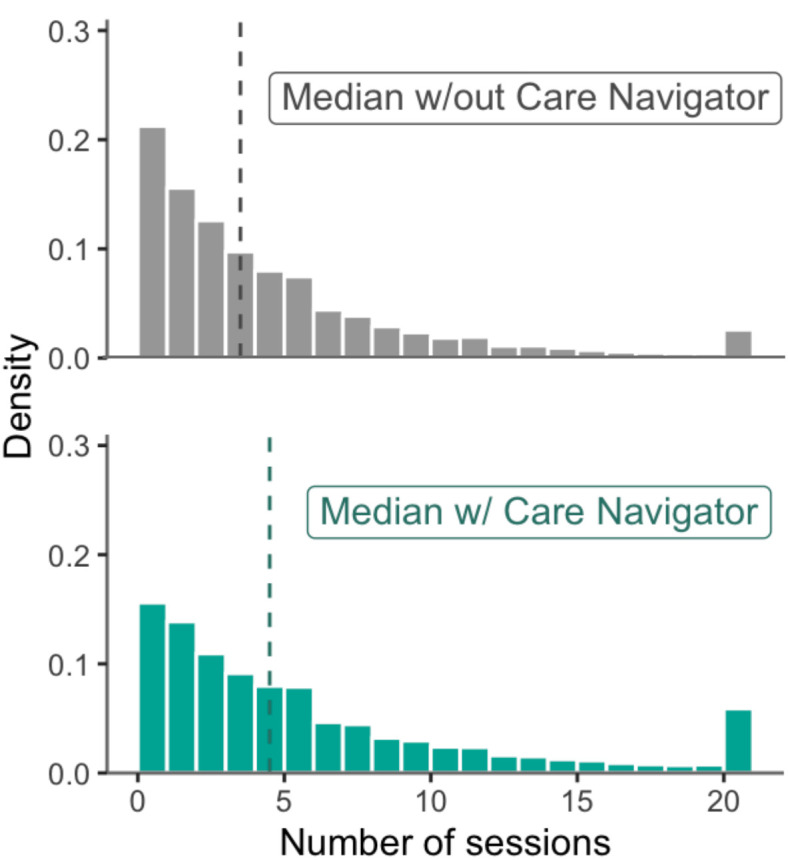
Number of treatment sessions with care navigation. Distribution of treatment sessions for participants without care navigation (top) compared to those using care navigation (bottom), including those attending over 20 sessions. Dashed lines indicate the median number of treatment sessions.

Participants using care navigation attended a median of 5 sessions (IQR, 2–7) compared to 4 sessions (IQR, 2–9) for those not using care navigation (Fig 3). Care navigation was associated with attending 36% more sessions (IRR, 1.36; 95% CI, 1.330–1.39, P < 0.001; S3 Table in S1 File), corresponding to a group estimate of 7.6 sessions (95% CI, 7.3–7.9) for those using care navigation and 5.2 sessions (95% CI, 5.0–5.3) for those who did not. In the care navigation group, each *additional* appointment with a Care Navigator was associated with an increase in the duration of care of 21% (IRR, 1.21; 95% CI, 1.19–1.23, P < 0.001), although the modal and median number of appointments with a care navigator was 1. Baseline severity and use of care navigation had a significant interaction effect on duration of care (IRR, 1.009; 95% CI, 1.005–1.013, P < 0.001), with participants using care navigation showing a greater increase in duration of care with increasing baseline severity. Participants of color had 5% shorter duration of care (IRR, 0.946; 95% CI, 0.923–0.969, P < 0.001), but the effect of care navigation on treatment duration interacted with identifying as a person of color (IRR, 1.102; 95% CI, 1.050–1.156, P < 0.001) such that White participants saw a slightly larger increase associated with care navigation, with their average number of sessions increased from 5.7 to 7.8 sessions and participants of color’s average number of sessions increased from 5.1 to 7.0 sessions. There was no interaction between care navigation, person of color, and baseline symptom severity (IRR, 1.000; 95% CI, 0.992–1.008, P = 0.983). When analyzed by specific racial groups, there was no interaction between race and care navigation on number of sessions attended relative to White participants (Ps > 0.079), except for middle eastern participants (n = 21), whose number of sessions attended was significantly more White participants (IIR, 2.15, 95% CI 1.05–4.49, P = 0.038).

Among participants who converted to care, scores on the PHQ-9 and GAD-7 were moderate prior to starting therapy (mean [SD], PHQ-9: 11.2 [6.2]; GAD-7: 10.3 [5.5]). Overall, participants experienced clinical improvements across treatment, shown both by a decrease in symptom severity by linear session number (PHQ-9: *b* = −0.51, P < 0.001; GAD-7 *b* = −0.35, P < 0.001) (S4 and S5 Tables in S1 File) and by the overall treatment effect given by multiplying the rate of change by the average number of sessions for those attending therapy alone (i.e., no care navigation group, 5.3 [95% CI, 5.2–5.4] sessions). This yielded a 2.66-point (95% CI, 2.49–2.83, *d* = 0.4) improvement in PHQ-9 scores and 2.06 points (95% CI, 1.92–2.21, *d* = 0.4) in GAD-7 scores. When comparing the clinical improvement for participants using care navigation compared to those using therapy alone, there was an interaction between care navigation use and linear session number for both PHQ-9 scores and GAD-7 scores, (PHQ-9: *b* = 0.17, P < 0.001; GAD-7: *b* = 0.11, P < 0.001), but there was no additional improvement in depression or anxiety for those using care navigation (PHQ-9: 0.19 points, 95% CI, −0.08–0.49, *d* = 0.03; GAD-7: 0.20 points, 95% CI, −0.02–0.43, *d* = 0.04).

**Fig 4:**
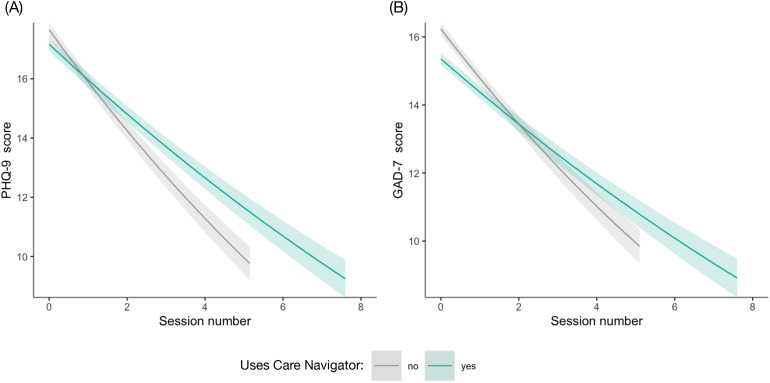
Clinical outcomes with care navigation. Clinical outcomes for (A) depression (PHQ-9) and (B) anxiety (GAD-7) for high severity participants. Error bands correspond to 95% CI.

Participants with high baseline severity (PHQ-9 or GAD-7 > 15 at baseline) showed a large overall treatment effect (PHQ-9: 6.40 points, 95% CI, 5.86–6.65, *d* = 1.7; GAD-7: 5.07 points, 95% CI, 4.57–5.65, *d* = 1.8) and critically, care navigation use was associated with an *additional* improvement of 0.93 points (95% CI, 0.11–1.75, *d* = 0.25) in depression symptoms and an additional 0.87 points (95% CI, 0.12–1.62, *d* = 0.3) in anxiety symptoms (Fig 4). There was no main effect of identifying as a person of color on PHQ-9 scores (*b* = 0.067, P = 0.844) nor GAD-7 scores (*b* = −0.181, P = 0.567), no interaction between identifying as a as person of color and session number (PHQ-9; *b* = −0.115, P = 0.083; GAD-7 *b* = −0.068, P = 0.262) nor between identifying as a person of color, session number, and care navigator (PHQ-9; *b* = −0.080, P = 0.559; GAD-7:*b* = −0.165, P = 0.190) (S6 and S7 Tables in S1 File). Thus, for high-severity participants, the overall clinical improvement and the effect of care navigation on such improvement was consistent for White participants and participants of color.

## Discussion

We conducted the largest-ever study on clinical care navigation in mental health as part of an employer-sponsored benefit program, involving over 35,000 more participants than previous studies.[18,26] Our primary objective was to explore the association between use of care navigation and therapy utilization as well as clinical improvements in depression and anxiety. A secondary objective was to determine whether these effects were consistent for people of color.

Among participants motivated to complete a mental health assessment—an inclusion criteria for both the care navigation and non-care navigation groups—those using care navigation had 7.1 times increased odds of starting therapy and attended 36% more sessions. Furthermore, the use of care navigation was associated with additional clinical improvements in patients with severe depression and anxiety, beyond those achieved through therapy alone. These findings align with those of a previous, smaller randomized controlled study, which showed that employees receiving care navigation and outreach were more likely than those receiving usual care to access mental health specialty treatment and to attend more appointments [18]. The high quality and strong positive outcomes observed in both the previous and current studies may stem from employing licensed mental health clinicians as care navigators, rather than peer advocates or non-licensed healthcare workers, which is more common [19]. All care navigators in this study held LCSW, LPC, LMFT or other licenses. Deeper expertise with mental healthcare may have enabled care navigators to provide better quality therapist recommendations and better advise and motivate participants with concerns about continuing care. Here, participants using care navigation had lower odds of dropping out after a single session.

From therapy alone, participants with high severity symptoms experienced a 6.40-point reduction in depression symptoms and 5.07-point reduction in anxiety symptoms, (*d*s >=1.7). Those using care navigation saw small additional improvements (*d*s>=0.25), even though they received no additional clinical treatment from care navigators. Previous studies have shown that care navigation is most effective with increasing severity, especially among those with severe mental illness [15–17]. Among all participants, care navigation was associated with increased conversion to care and more sessions of therapy, but no additional improvements beyond the treatment effect. This aligns with earlier findings that care navigation interventions are less effective for individuals with low symptom severity [18].

For both utilization and clinical measures, using care navigation was associated with better outcomes for both White participants and participants of color. When comparing specific racial demographics, the association between care navigation and conversion to care, session attendance, and total sessions was largely consistent with that observed for White participants, underscoring the platform’s broadly equitable impact when navigators apply culturally responsive, inclusive practices. However, there were some deviations: Compared to White participants using care navigation, Native American members using care navigation exhibited significantly lower odds of converting to care, Mixed Race participants using care navigation showed higher odds of attending multiple sessions, and Middle Eastern members using care navigation attended more total sessions. Although most of these differences emerged in small subgroups and did not follow an apparent pattern, we cannot rule out that care navigators interacted differently with these groups, and while the foundational diversity and cultural-competence training of care navigators promotes generally equitable engagement across populations, small-sample variations may reflect unique cultural dynamics or persistent access barriers in specific communities, highlighting the importance of ongoing, targeted training and adaptive strategies.

Overall, just as community-based or peer care navigation [15–17] improves access to primary care clinics for people of color, [25] our results suggest that a clinical care navigation program—especially when implemented in a sustained, scalable manner, such as part of an employer-sponsored benefit—may support more equitable patterns of initiation and retention across racial groups by helping individuals understand and navigate complicated care pathways.

Although our analysis was limited to the 2018–2023 window, several additional evaluations of the mental health benefit have been conducted, showing consistent clinical outcomes [31] and financial returns for employers [32], and care navigation remains a key component of the program design. By driving over seven‐fold higher therapy initiation, reducing dropout, and extending total treatment exposure, remote navigation may therefore set patients on a trajectory toward longer‐term mental health stability and ongoing reductions in medical and productivity costs. Longer follow‐up studies will be important to confirm whether these downstream advantages persist once active navigation concludes.

### Strengths and limitations

This study, the largest of its kind on care navigation, included a diverse and realistic sample of 42% people of color from 2,045 employers across all US states, with a baseline comorbidity rate similar to the general population. [33,34] However, the non-randomized design means that we cannot rule out other potential causes of group differences besides care navigation. For example, self‐selection into care navigation—whereby the patients most motivated for change [35] both choose to enroll and are predisposed to seek, adhere to, and benefit from services—creates a confounding bias that could inflate any apparent effect of navigation on conversion, retention, and clinical improvement. In practical terms, comparing outcomes for patients who use care navigation with those who don’t mixes the program’s effects with patients’ own motivation and engagement, potentially inflating estimates of care navigation’s true impact. Although we matched the two groups on baseline severity, optimism for treatment, age, person of color status, gender, number of sponsored therapy sessions, and care navigation recommendation, this study lacks a direct measure of motivation, and as a retrospective cohort study, we cannot disentangle the pure contribution of care navigation from the underlying commitment of patients. Consequently, the effects associated with care navigation may be due in part to motivation, and these results may not generalize to less- or differently motivated populations. Additionally, engagement levels could vary among specific racial groups or comorbidities, and our study focuses on only those who have access to the mental health benefit (employees and their dependents) and may not generalize to unemployed populations. Regardless of whether they converted to care, all participants were enrolled in the benefit, and therefore may correspond to a subset of employees for whom mental health is more relevant.

### Clinical considerations

Care navigation is a promising approach in mental healthcare to improve access and treatment outcomes. In this retrospective cohort study, participants were over seven times more likely to begin therapy, less likely to drop out of therapy after a single session, and attended more therapy sessions when using a clinical care navigation program within an employer-sponsored mental health benefit. Such a care navigation solution may be a cost‑effective way to maximize the impact of mental health programs in general. Mental health programs deliver strong financial returns [32] (i.e., through health plan cost savings) when employees both enroll and remain engaged long enough to see benefits. While care navigation adds an additional upfront expense beyond basic behavioral healthcare, its ability to get more people into care and keep them engaged may contribute to downstream economic gains. For the highest-risk participants, using care navigation was also associated with significant reductions in depression and anxiety symptoms. These effects were consistent for White participants and participants of color. Although this study is limited to employer-sponsored mental healthcare, the care navigation model generally is associated with increased access to and retention of health insurance and thus suggests that care navigation may be a promising approach to address barriers to mental healthcare even among those less resourced than those in the present study.

## Supporting information

S1 File Supporting information file. (DOCX)
