# Supplementary material for: A remote care navigation solution associated with improved utilization and outcomes of mental healthcare: A nationwide cohort study in the USA
Source: PLoS One. 2025 Sep 18;20(9):e0331454. doi: 10.1371/journal.pone.0331454 (PMC12445557; doi:10.1371/journal.pone.0331454)
Supplement: S1 File — (DOCX) [file pone.0331454.s001.docx]

**Online Material**

**Methods**

**Program design**

We used data from an employer-sponsored digital mental health benefit (Spring Health; Spring Care Inc). The program has previously been demonstrated to improve both clinical and financial outcomes[^24^](https://www.zotero.org/google-docs/?RB7W8B), and was implemented by 2,045 employers across all 50 states in the US. The program incorporates several evidence-based components, including a digital mental health assessment tool, telephone and video appointments with care navigators who help individuals enrolled in the benefit find appropriate care, online cognitive behavioral therapy resources, free or low-cost access to psychotherapy and medication management through video or in-person sessions, and a symptom monitoring system to support measurement-based care. Individuals electively complete a series of assessments of common mental health problems (e.g. stress, anxiety, sleeping, eating, or relationship problems), including the Patient Health Questionnaire-9 for depression (PHQ-9; range, 0-27)[^25,26^](https://www.zotero.org/google-docs/?Mgzqnd) and the Sheehan Disability Scale[^27^](https://www.zotero.org/google-docs/?27oM8d) for functional impairment, with additional questionnaires triggered pending screening responses. They are asked how optimistic they feel that therapy can help them and choose regular intervals to complete follow-up assessments, with the default being every 2 weeks.

After completing the initial assessment, individuals receive a personalized care plan and can schedule appointments with care navigators, therapists, medication managers, or coaches. Care plans vary based on assessment results and symptom severity, which are used to determine risk for mental health problems. Overall risk level was set by the maximum risk level of any of the assessments. On the PHQ-9 for depression and the Generalized Anxiety Disorder-7[^28^](https://www.zotero.org/google-docs/?d6eit5), scores 15 or over are categorized as high-risk, from 10-14 as moderate risk, from 5 to 9 as moderate-low risk, and under 5 as low risk. Care navigation is always recommended within the platform for high-risk individuals, but the recommendation differs for those with less severe mental health problems, depending on when the individual enrolled in the program and on their specific issues. Regardless of explicit recommendation by the platform, care navigation is available to all enrolled individuals and is delivered over the phone or video. Outpatient care appointments (i.e. with a therapist, coach, or medication manager) are delivered via video or in-person. Care navigators have masters degree–level clinical licenses, therapists have masters- or doctoral-level licenses, medication managers are medical doctors or doctors of osteopathic medicine. All clinicians have had at least 3 years of experience post-supervision before joining the network.

Individuals could schedule unlimited free appointments with their care navigators. Depending on the benefit design offered by their employer, Individuals could schedule appointments with a program therapist, a coach, and/or medication manager, for which the employer would either cover all or a limited number of visits, or the Individuals could pay for the appointment themselves.

**Measures**

When included as an interaction term (e.g. with the effect of care navigation), the highest of the two baseline severity scores was used. When assessing clinical improvement, depression and anxiety symptoms were modeled separately and not as comorbid factors. Response to prior treatment, treatment resistance, or current psychotropic medication usage was not measured.

**Results**

**Employer Characteristics**

By total employees, the largest industries represented were healthcare (22%), technology (18%), manufacturing (8%), and education (7%). Employers also differed in the number of sponsored sessions they offered: 39% of employees had access to six sponsored sessions, 21% had access to twelve, 14% had access to eight, and 12% had access to four, with the remainder having access to two to unlimited sessions.

**Appointment Times**

Median time to first available care navigation appointment was 2.0 days (IQR, 0.8-4.0 days) and 1.1 days (IQR, 1.0-1.7 days) for first available psychotherapy appointment. Of those participants using care navigation, 50% had their initial appointment with a Care Navigator within 10 days of their initial assessment, 75% by 47 days, and 90% by 146 days.

**Impact of recommendations for care navigation**

Participants who used care navigation had much higher odds of starting therapy than those who did not, among participants not recommended care navigation (OR, 6.82; 95% CI, 3.04–15.30, P<0.001) and those recommended care navigation (OR, 10.51; 95% CI, 5.98–18.46, P<0.001). Participants who used care navigation had greater odds of attending more than one therapy session (i.e. less likely to drop out after a single session) than those who did not, among participants not recommended care navigation (OR, 1.64; 95% CI, 1.51–1.77, P<0.001) but not those recommended care navigation (OR, 1.21; 95% CI, 0.96–1.54, P=0.112).

**Impact of employer and care navigator random effect terms**

Including random effect terms for employer and care navigator produced a better fit compared to models without these terms, as compared by AIC and BIC, for all models (except the negative binomial model, which failed to converge when these terms were included). The primary effects of interest (main effect of care navigation, interaction between care navigation use and baseline severity, and the interaction between care navigation and person of color) were comparable between the models with and without employer and care navigator random effect terms. When predicting conversion to care, including these random effects produced a wider 95% confidence interval for the main effect of care navigation on conversion to care (OR, 7.10, 95% CI 3.936–15.00) compared to the model without (OR, 7.22, 95% CI 6.80–7.66).

**S1 Table 1. Logistic regression results for converting to care.**

|  | **Converted to care** | | |
| --- | --- | --- | --- |
| *Predictors* | *Odds Ratio* | *95% CI* | *p* |
| (Intercept) | 2.977 | 1.822 – 4.862 | **<0.001** |
| uses care navigation | 7.101 | 3.361 – 15.001 | **<0.001** |
| baseline severity | 1.012 | 1.007 – 1.017 | **<0.001** |
| person of color | 1.024 | 0.968 – 1.083 | 0.412 |
| recommended care navigation | 0.747 | 0.690 – 0.809 | **<0.001** |
| treatment optimism | 1.107 | 1.093 – 1.121 | **<0.001** |
| gender | 0.936 | 0.884 – 0.991 | **0.024** |
| age | 0.987 | 0.984 – 0.989 | **<0.001** |
| employer-sponsored sessions | 1.074 | 1.045 – 1.105 | **<0.001** |
| uses care navigation × baseline severity | 0.975 | 0.965 – 0.985 | **<0.001** |
| uses care navigation × person of color | 0.919 | 0.809 – 1.044 | 0.193 |
| baseline severity × person of color | 0.996 | 0.987 – 1.005 | 0.354 |
| uses care navigation × baseline severity × person of color | 0.996 | 0.975 – 1.017 | 0.688 |
| **Random Effects** | | | |
| σ^2^ | 3.29 | | |
| τ_00_ _employer_id_ | 0.54 | | |
| τ_00_ _care_navigator_id_ | 0.14 | | |
| ICC | 0.17 | | |
| N _employer_id_ | 299 | | |
| N _care_navigator_id_ | 106 | | |
| Observations | 36132 | | |
| Marginal R^2^ / Conditional R^2^ | 0.199 / 0.337 | | |

**S2 Table. Logistic regression results for attending multiple sessions of therapy.**

|  | **Attended multiple sessions** | | |
| --- | --- | --- | --- |
| *Predictors* | *Odds Ratio* | *95% CI* | *p* |
| (Intercept) | 4.929 | 4.640 – 5.235 | **<0.001** |
| uses care navigation | 1.572 | 1.463 – 1.688 | **<0.001** |
| baseline severity | 1.007 | 1.000 – 1.013 | **0.035** |
| person of color | 0.938 | 0.874 – 1.008 | 0.081 |
| recommended care navigation | 0.829 | 0.746 – 0.921 | **<0.001** |
| treatment optimism | 1.032 | 1.013 – 1.050 | **0.001** |
| gender | 0.981 | 0.908 – 1.059 | 0.619 |
| age | 1.002 | 0.999 – 1.006 | 0.229 |
| employer-sponsored sessions | 1.050 | 1.030 – 1.071 | **<0.001** |
| uses care navigation × baseline severity | 1.017 | 1.005 – 1.029 | **0.005** |
| uses care navigation × person of color | 1.016 | 0.884 – 1.167 | 0.828 |
| baseline severity × person of color | 1.009 | 0.997 – 1.021 | 0.132 |
| uses care navigation × baseline severity × person of color | 1.015 | 0.991 – 1.039 | 0.225 |
| **Random Effects** | | | |
| σ^2^ | 3.29 | | |
| τ_00_ _employer_id_ | 0.05 | | |
| τ_00_ _care_navigator_id_ | 0.00 | | |
| N _employer_id_ | 287 | | |
| N _care_navigator_id_ | 106 | | |
| Observations | 22934 | | |
| Marginal R^2^ / Conditional R^2^ | 0.024 / NA | | |

**S3 Table. Negative binomial regression results for duration of therapy (# sessions)**

|  | **Number of sessions** | | |
| --- | --- | --- | --- |
| *Predictors* | *Incidence Rate Ratios* | *95% CI* | *p* |
| (Intercept) | 6.097 | 6.027–6.347 | **<0.001** |
| uses care navigation | 1.362 | 1.330–1.394 | **<0.001** |
| person of color | 0.900 | 0.879–0.921 | **<0.001** |
| baseline severity | 1.002 | 1.000–1.004 | 0.083 |
| recommended care navigation | 0.922 | 0.889–0.956 | 0.134 |
| treatment optimism | 1.021 | 1.015–1.027 | **<0.001** |
| gender | 0.970 | 0.946–0.996 | **0.023** |
| age | 0.998 | 0.997–0.999 | **0.002** |
| employer-sponsored sessions | 1.021 | 1.017–1.025 | **<0.001** |
| uses care navigation × person of color | 1.007 | 0.961–1.055 | 0.778 |
| uses care navigation × baseline severity | 1.005 | 1.001–1.009 | **0.020** |
| baseline severity × person of color | 1.000 | 0.996–1.004 | 0.939 |
| uses care navigation × baseline severity × person of color | 1.000 | 0.992–1.008 | 0.986 |
| Observations | 23556 | | |
| R^2^ Nagelkerke | 0.058 | | |

**S4 Table. Mixed effects regression results for PHQ-9 scores**

|  | **PHQ-9 score** | | |
| --- | --- | --- | --- |
| *Predictors* | *Estimates* | *std. Error* | *p* |
| (Intercept) | 8.117 | 0.123 | **<0.001** |
| session number | -0.508 | 0.014 | **<0.001** |
| uses care navigation | 0.032 | 0.129 | 0.803 |
| person of color | 0.117 | 0.124 | 0.345 |
| gender | -0.382 | 0.100 | **<0.001** |
| age | -0.054 | 0.004 | **<0.001** |
| recommended care navigation | 4.381 | 0.141 | **<0.001** |
| treatment optimism | -0.025 | 0.024 | 0.288 |
| session number^2^ | 0.027 | 0.001 | **<0.001** |
| session number^3^ | -0.000 | 0.000 | **<0.001** |
| total visits covered | -0.024 | 0.040 | 0.546 |
| uses care navigation × session number | 0.166 | 0.029 | **<0.001** |
| uses care navigation × session number^2^ | -0.017 | 0.002 | **<0.001** |
| uses care navigation × session number^3^ | 0.000 | 0.000 | **<0.001** |
| person of color × session number | 0.007 | 0.028 | 0.804 |
| person of color × uses care navigation | 0.360 | 0.247 | 0.145 |
| person of color × uses care navigation × session number | -0.039 | 0.058 | 0.499 |
| **Random Effects** | | | |
| σ^2^ | 7.89 | | |
| τ_00_ _member_id_ | 21.76 | | |
| τ_00_ _customer_name_ | 1.24 | | |
| τ_00_ _cn_provider_id_ | 0.00 | | |
| τ_11_ _member_id.session_number_ | 0.46 | | |
| ρ_01_ _member_id_ | -0.20 | | |
| N _member_id_ | 13403 | | |
| N _cn_provider_id_ | 68 | | |
| N _employer_id_ | 204 | | |
| Observations | 45291 | | |
| Marginal R^2^ / Conditional R^2^ | 0.433 / NA | | |

**S5 Table. Mixed effects regression results for GAD-7 scores**

|  | **GAD-7 score** | | |
| --- | --- | --- | --- |
| *Predictors* | *Estimates* | *std. Error* | *p* |
| (Intercept) | 7.578 | 0.128 | **<0.001** |
| session number | -0.353 | 0.009 | **<0.001** |
| uses care navigation | -0.244 | 0.251 | 0.331 |
| person of color | -0.446 | 0.098 | **<0.001** |
| gender | -0.680 | 0.073 | **<0.001** |
| age | -0.076 | 0.003 | **<0.001** |
| recommended care navigation | 3.354 | 0.097 | **<0.001** |
| treatment optimism | 0.083 | 0.017 | **<0.001** |
| session number^2^ | 0.014 | 0.000 | **<0.001** |
| session number^3^ | -0.000 | 0.000 | **<0.001** |
| total visits covered | -0.017 | 0.024 | 0.470 |
| uses care navigation × session number | 0.105 | 0.018 | **<0.001** |
| uses care navigation × session number^2^ | -0.015 | 0.001 | **<0.001** |
| uses care navigation × session number^3^ | 0.000 | 0.000 | **<0.001** |
| person of color × session number | 0.034 | 0.018 | 0.058 |
| person of color × uses care navigation | 0.198 | 0.196 | 0.311 |
| person of color × uses care navigation × session number | -0.017 | 0.037 | 0.653 |
| **Random Effects** | | | |
| σ^2^ | 6.55 | | |
| τ_00_ _member_id_ | 18.65 | | |
| τ_00_ _customer_name_ | 0.57 | | |
| τ_00_ _cn_provider_id_ | 0.05 | | |
| τ_11_ _member_id.outcome_session_number_ | 0.36 | | |
| ρ_01_ _member_id_ | -0.21 | | |
| N _member_id_ | 22573 | | |
| N _cn_provider_id_ | 105 | | |
| N _employer_id_ | 287 | | |
| Observations | 84748 | | |
| Marginal R^2^ / Conditional R^2^ | 0.160 / 0.787 | | |

**S6 Table. Mixed effects regression for PHQ-9 scores for high severity participants (PHQ-9 baseline >15)**

|  | **PHQ-9 score** | | |
| --- | --- | --- | --- |
| *Predictors* | *Estimates* | *std. Error* | *p* |
| (Intercept) | 12.352 | 0.184 | **<0.001** |
| session number | -1.102 | 0.033 | **<0.001** |
| uses care navigation | 0.976 | 0.351 | **0.006** |
| person of color | 0.067 | 0.341 | 0.844 |
| gender | -0.005 | 0.163 | 0.976 |
| age | -0.004 | 0.007 | 0.602 |
| recommended care navigation | 0.409 | 0.170 | **0.016** |
| treatment optimism | -0.148 | 0.035 | **<0.001** |
| session number^2^ | 0.033 | 0.001 | **<0.001** |
| session number^3^ | -0.001 | 0.000 | **<0.001** |
| total visits covered | 0.003 | 0.032 | 0.919 |
| uses care navigation × session number | 0.301 | 0.067 | **<0.001** |
| uses care navigation × session number^2^ | -0.026 | 0.003 | **<0.001** |
| uses care navigation × session number^3^ | 0.000 | 0.000 | **<0.001** |
| person of color × session number | -0.115 | 0.067 | 0.083 |
| person of color × uses care navigation | 0.371 | 0.697 | 0.595 |
| person of color × uses care navigation × session number | 0.080 | 0.138 | 0.559 |
| **Random Effects** | | | |
| σ^2^ | 13.07 | | |
| τ_00_ _member_id_ | 11.58 | | |
| τ_00_ _customer_name_ | 0.30 | | |
| τ_00_ _cn_provider_id_ | 0.00 | | |
| τ_11_ _member_id.outcome_session_number_ | 1.08 | | |
| ρ_01_ _member_id_ | 0.22 | | |
| N _member_id_ | 4624 | | |
| N _cn_provider_id_ | 96 | | |
| N _employer_id_ | 204 | | |
| Observations | 16878 | | |
| Marginal R^2^ / Conditional R^2^ | 0.698 / NA | | |

**S7 Table. Mixed effects regression results for GAD-7 for high severity participants (GAD-7 baseline >15)**

|  | **GAD-7 score** | | |
| --- | --- | --- | --- |
| *Predictors* | *Estimates* | *std. Error* | *p* |
| (Intercept) | 11.634 | 0.163 | **<0.001** |
| session number | -0.886 | 0.030 | **<0.001** |
| uses care navigation | 0.143 | 0.324 | 0.659 |
| person of color | -0.181 | 0.316 | 0.567 |
| gender | -0.008 | 0.136 | 0.952 |
| age | -0.018 | 0.006 | **0.002** |
| recommended care navigation | 0.526 | 0.143 | **<0.001** |
| treatment optimism | -0.026 | 0.030 | 0.386 |
| session number^2^ | 0.028 | 0.001 | **<0.001** |
| session number^3^ | -0.000 | 0.000 | **<0.001** |
| total visits covered | -0.009 | 0.024 | 0.703 |
| uses care navigation × session number | 0.229 | 0.062 | **<0.001** |
| uses care navigation × session number^2^ | -0.024 | 0.002 | **<0.001** |
| uses care navigation × session number^3^ | 0.000 | 0.000 | **<0.001** |
| person of color × session number | -0.068 | 0.061 | 0.262 |
| person of color × uses care navigation | -0.609 | 0.646 | 0.347 |
| person of color × uses care navigation × session number | -0.165 | 0.126 | 0.190 |
| **Random Effects** | | | |
| σ^2^ | 9.49 | | |
| τ_00_ _member_id_ | 8.01 | | |
| τ_00_ _customer_name_ | 0.10 | | |
| τ_00_ _cn_provider_id_ | 0.00 | | |
| τ_11_ _member_id.outcome_session_number_ | 1.00 | | |
| ρ_01_ _member_id_ | 0.21 | | |
| N _member_id_ | 4625 | | |
| N _cn_provider_id_ | 96 | | |
| N _employer_id_ | 204 | | |
| Observations | 17127 | | |
| Marginal R^2^ / Conditional R^2^ | 0.684 / NA | | |
